# Causal relationship and shared genetic pathways between diabetic kidney disease and cognitive impairment: a Mendelian randomization study

**DOI:** 10.1080/0886022X.2025.2525471

**Published:** 2025-07-01

**Authors:** Ke Yu, Yanqing Chi, Qian Wang, Yongzhe Chen, Ning Han, Ying Li

**Affiliations:** a Department of Nephrology, Hebei Medical University Third Hospital, Shijiazhuang City, China; b Hebei Key Laboratory of Diabetic Kidney Disease, Shijiazhuang City, China

**Keywords:** Diabetic kidney disease, cognitive impairment, Mendelian randomization, genetic correlation, genome-wide association study

## Abstract

**Background:**

Diabetic kidney disease (DKD) may increase cognitive impairment (CI) risk, but causal evidence remains limited. We aimed to investigate this causality and elucidate underlying genetic mechanisms.

**Methods:**

Bidirectional two-sample Mendelian randomization (MR) was performed using summary statistics from four DKD genome-wide association studies (GWAS) and a large-scale cognitive function GWAS. Sensitivity analyses (heterogeneity, pleiotropy, leave-one-out, and Steiger directionality tests) confirmed the robustness of the results. Multivariable MR was used to adjust for potential confounders. Genetic correlation was assessed via linkage disequilibrium score regression (LDSC). Shared loci and pathways were identified through colocalization and functional enrichment analyses.

**Results:**

DKD significantly increased cognitive decline risk (inverse-variance weighted (IVW) OR range: 0.55–0.88; all *p* < .05), with consistent results across sensitivity analyses. Multivariable MR confirmed that the association was independent of confounders. Significant genetic correlations were observed (LDSC rg = 0.072–0.201). Colocalization identified six shared risk loci (posterior probability for H4 (PP.H4) > 0.90). Enriched pathways included ribosomal function, mitochondrial oxidative phosphorylation, and neurodegeneration.

**Conclusions:**

This study provides evidence supporting a causal relationship and genetic correlation between DKD and CI, while also identifying shared genetic features and biological pathways that may contribute to their association.

## Graphical Abstract

**Figure:**
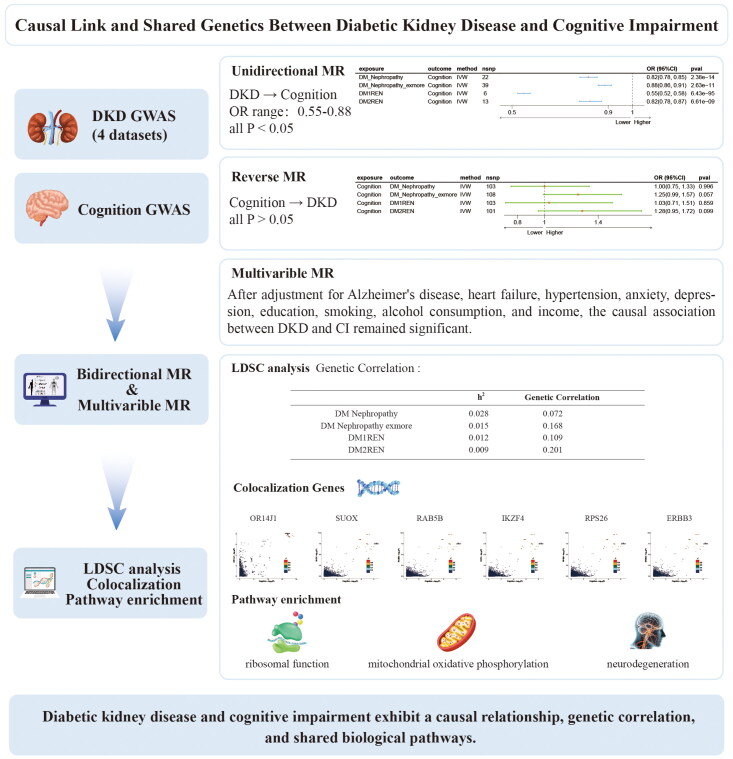

Causal link and shared pathways between diabetic kidney disease and cognitive impairment.

## 1. Introduction

The global prevalence of diabetes has risen dramatically, with 537 million cases reported in 2021 and projections reaching 784 million by 2045 [1]. This epidemic has driven a parallel increase in diabetic kidney disease (DKD), which affects approximately 40% of diabetes patients and represents the leading cause of chronic kidney disease (CKD) worldwide [2]. Emerging evidence reveals that DKD is not only a renal complication but also significantly impacts neurological health, with patients exhibiting a markedly elevated risk of cognitive impairment (CI) [3–5].

The relationship between DKD and CI has become a growing public concern. Research has shown that patients with DKD exhibit lower cognitive performance in areas such as attention, language ability, executive function, delayed recall, and other cognitive domains, with these deficits correlating with the severity of kidney dysfunction [6]. This DKD-CI association carries important clinical implications, as CI further exacerbates poor outcomes in DKD patients, increasing risks of cardiovascular events and mortality [7,8]. Both diabetes and renal dysfunction are recognized as risk factors for CI [9–11]. However, the causal relationship between DKD and CI remains unclear, and the potential influence of confounders and reverse causation has yet to be fully addressed. Additionally, the shared genetic basis underlying the association between DKD and CI is poorly understood.

Mendelian randomization (MR) analysis is a powerful method that uses genetic variants as instrumental variables (IVs) to infer causal relationships between exposures and outcomes. This approach is often referred to as a ‘natural randomized controlled trial’ because it leverages the random allocation of genetic variants, such as single nucleotide polymorphisms (SNPs), to minimize bias from confounding factors or reverse causation [12,13]. MR has gained widespread recognition as a robust tool for investigating causal relationships between exposures and outcomes. Linkage disequilibrium score regression (LDSC) is another statistical method that estimates genetic correlations using summary statistics from genome-wide association studies (GWAS). Colocalization analysis further strengthens genetic findings by identifying shared genetic variants associated with both exposure and outcome, thereby providing stronger evidence for causal relationships.

In this study, we employed MR analysis to explore the causal relationship between DKD and CI and to identify shared genetic loci and biological pathways underlying their comorbidity. The findings aim to provide a foundation for the prevention and treatment of CI in patients with DKD, offering new insights into the interplay between DKD and cognitive health.

## 2. Materials and methods

**Figure 1.**
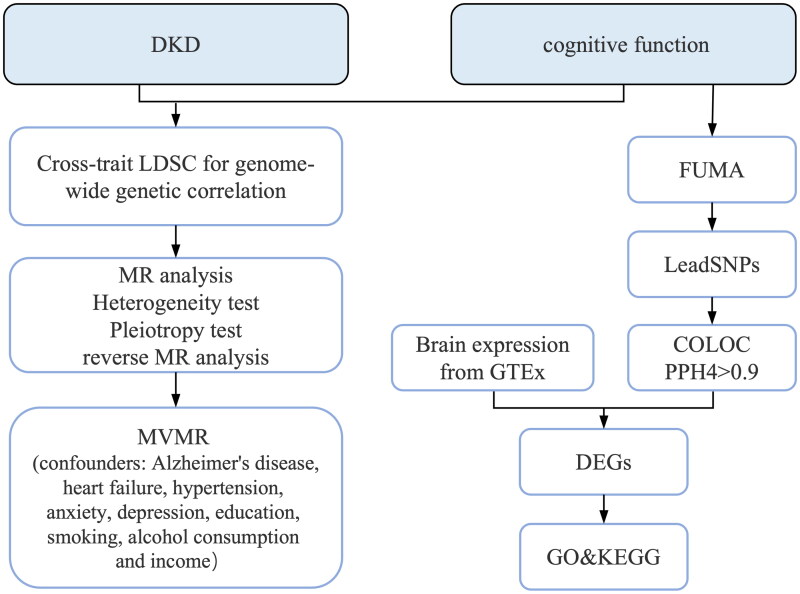
Schematic overview of the study design and experimental workflow.

The study design is illustrated in Figure 1. All data used in this study were obtained from previously published studies or publicly accessible databases, eliminating the need for additional ethical approval.

### 2.1. Data sources

The GWAS datasets for DKD were sourced from the publicly available database of the FinnGen project [14], which comprises participants of European ancestry. Detailed information on these datasets is provided in Supplementary Table 1.

The cognitive function dataset was derived from a meta-analysis of 1.1 million individuals conducted by Lee et al. [15], which exclusively included data from European populations. A Manhattan plot summarizing the cognitive function GWAS results is provided in Supplementary Figure 1.

Data on potential confounders, including Alzheimer’s disease, hypertension, heart failure, income, education, anxiety, depression, smoking, and alcohol consumption, were sourced from the IEU Open GWAS database (https://gwas.mrcieu.ac.uk/). Dataset IDs and sample sizes are listed in Supplementary Table 2.

### 2.2. Selection of instrumental variables

SNPs were employed as IVs, which were required to satisfy three key assumptions [16]: (a) *Relevance*: IVs must be strongly associated with the exposure; (b) *Independence*: IVs must not be associated with confounders; and (c) *Exclusivity*: IVs must influence the outcome solely through the exposure.

A systematic approach was employed to select the most appropriate IVs for both forward and reverse MR analyses, with the following criteria: (a) the significance threshold for all IVs was set at *p* < 5 × 10^−8^; (b) to avoid bias from weak IVs, SNPs with an *F* statistic >10 were selected; (c) only loci with a minor allele frequency (MAF) greater than 1% were retained; (d) linked disequilibrium sites were removed, the linkage disequilibrium (LD) coefficient (*r*^2^) was set to 0.001, and the width of the LD region was restricted to 10,000 kb.

### 2.3. Statistical analysis

#### 2.3.1. FUMA and S-LDSC analysis

The functional mapping and annotation (FUMA) [17] was utilized to annotate cognition-related GWAS data based on the positional and functional information of SNPs. Significant SNPs (*p* < 5 × 10^−8^, LD *r*^2^ < 0.1) associated with cognition were identified as risk loci and mapped to genes. Subsequently, the enriched sites of cognition risk loci were obtained at the tissue level according to gene expression levels in 54 tissues of GTEx V8. Stratified linkage disequilibrium score regression (S-LDSC) [18] was applied to cognition GWAS summary statistics to identify genomic functional annotations most relevant to common variant signals.

#### 2.3.2. Mendelian randomization

To assess the causal relationship between exposure and outcome, the following MR methods were employed: inverse-variance weighted (IVW) [19], weighted median [20], MR-Egger [21], simple mode [22], and weighted mode [23]. The IVW method served as the primary approach, while the other methods provided complementary evidence to validate the robustness of the findings. Sensitivity analyses were conducted to evaluate the robustness of the results, including Cochran’s *Q* test for heterogeneity, pleiotropy test (MR-Egger and MR-PRESSO), leave-one-out analysis, and Steiger directionality testing.

#### 2.3.3. Multivariate Mendelian randomization (MVMR)

MVMR analysis was used to determine the direct effect of exposure on outcome after adjusting for confounders. Potential confounders included Alzheimer’s disease, heart failure, hypertension, anxiety, depression, education, smoking, alcohol consumption, and income. These covariates were selected based on their established associations with both DKD and CI in previous epidemiological studies, and their potential to influence the genetic instruments used in our analysis. The causal effect was evaluated using the IVW method after adjusting for these confounders.

#### 2.3.4. Genetic correlation analysis

Linkage disequilibrium score regression was employed to estimate SNP-based heritability (*h*^2^). This method quantifies genetic correlations directly from GWAS summary statistics by modeling the relationship between test statistics and LD patterns. Given the large sample sizes of the DKD and cognition GWAS datasets, test statistic inflation was attributed to polygenic effects.

#### 2.3.5. Colocalization and enrichment analysis

Colocalization analysis was performed to identify shared genetic variants between exposure and outcome, strengthening the genetic study results. Lead SNPs were identified using FUMA, and the Coloc software [24] was applied to explore whether DKD and cognition share causal genetic variants within a 1 MB region upstream and downstream of the lead SNPs. The following hypotheses were tested: *H0*: No causal effect; *H1*: Causal effect on DKD only; *H2*: Causal effect on cognitive function only; *H3*: Causal effect on both DKD and cognitive function, but driven by different genetic variants; *H4*: Causal effect on both DKD and cognitive function, driven by the same genetic variant (colocalization sites). A posterior probability for H4 (PP.H4) >0.9 was used to identify genes with significant colocalization effects.

The file ‘GTEx Analysis 2017-06-05 v8 RSEMv1.3.0 transcript tpm.gct.gz’, obtained from the GTEx Analysis V8 dataset [25], was employed to investigate the impact of colocalization gene expression on brain function. The GTEx brain dataset was divided according to the upper quartile (high) and the lower quartile (low) of colocalization gene expression levels. Differentially expressed genes (DEGs) (*p*.adjust <.05 and |log FC| ≥2) were subjected to Gene Ontology (GO) and Kyoto Encyclopedia of Genes and Genomes (KEGG) pathway enrichment analyses to identify relevant biological processes, molecular functions, cellular components, and metabolic pathways.

## 3. Results

### 3.1. FUMA and S-LDSC analysis

**Figure 2.**
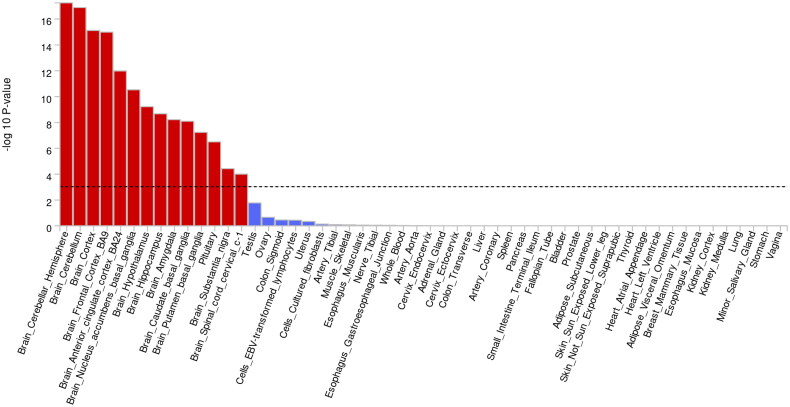
The FUMA analysis revealed significant tissue enrichment of cognition-related GWAS signals, primarily localized in 14 distinct neural structures, including brain cerebellar hemisphere, brain cerebellum, brain cortex, brain frontal cortex BA9, brain anterior cingulate cortex BA24, brain nucleus accumbens basal ganglia, brain hypothalamus, brain hippocampus, brain amygdala, brain caudate basal ganglia, brain putamen basal ganglia, pituitary, brain substantia nigra, and brain spinal cord cervical c-1.

The FUMA analysis revealed that the cognition-related GWAS was predominantly enriched in 14 central nervous system tissues, including the brain cerebellar hemisphere, brain cerebellum, and brain cortex (Figure 2). Additionally, S-LDSC identified significant enrichment for 62 annotation categories (FDR-corrected *p* < .05; Supplementary Table 3).

### 3.2. Mendelian randomization

**Figure 3.**
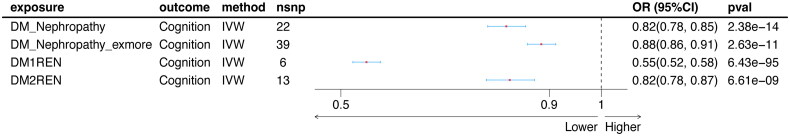
Forest plot displaying the causal effect of diabetic kidney disease on cognitive function from inverse-variance weighted Mendelian randomization analysis. IVW: inverse-variance weighted; nSNP: the number of SNPs used in the MR analysis; OR: odds ratio; 95% CI: 95% confidence interval; pval: *p* value.

MR analysis was conducted to investigate the causal relationship between DKD and cognitive function, using DKD as the exposure and cognitive function as the outcome. The analysis incorporated four independent DKD datasets, and the detailed results are presented in Supplementary Table 4. The MR analysis estimates demonstrated that DKD significantly increased the risk of impaired cognitive function, with odds ratios (ORs) below 1 across all datasets (range: 0.55–0.88, *p* < .001 for all). Specifically, the IVW method demonstrated: DM nephropathy: OR = 0.82 (95% CI: 0.78–0.85, *p* = 2.38 × 10^−14^); DM nephropathy exmore: OR = 0.88 (95% CI: 0.86–0.91, *p* = 2.63 × 10^−11^); DM1REN: OR = 0.55 (95% CI: 0.52–0.58, *p* = 6.43 × 10^−95^); DM2REN: OR = 0.82 (95% CI: 0.78–0.87, *p* = 6.61 × 10^−9^) (Figure 3). These consistent findings across all datasets suggest that DKD is causally associated with cognitive decline, thereby potentially increasing the risk of CI in affected individuals.

### 3.3. Sensitivity analysis

Sensitivity analyses consistently supported the robustness of our causal inferences. Cochran’s *Q* test for heterogeneity results indicated no significant heterogeneity across the IVs (*p* > .05; Table 1). Both the MR-Egger intercept test and the MR-PRESSO test revealed no evidence of horizontal pleiotropy (*p* > .05; Supplementary Table 5), suggesting that the IVs primarily influenced the outcome through the exposure. Leave-one-out sensitivity analysis further confirmed the robustness of the causal estimates, demonstrating that no single IV disproportionately drove the observed associations (Supplementary Figure 2). The Steiger directionality test confirmed the correct exposure to outcome causal direction (all *p* < .05), with genetic instruments explaining significantly greater variance in the exposure than in the outcome (Supplementary Table 6).

**Table 1.** Results of heterogeneity tests for instrumental variables.

Exposure	Outcome	Method	*Q*	*Q* df	*Q* pval
DM nephropathy	Cognition	MR-Egger	15.466	20	0.749
		IVW	29.590	21	0.101
DM nephropathy exmore		MR-Egger	40.251	37	0.328
		IVW	42.545	38	0.282
DM1REN		MR-Egger	0.608	4	0.962
		IVW	2.742	5	0.740
DM2REN		MR-Egger	10.233	11	0.510
		IVW	10.971	12	0.531

IVW: inverse variance weighted.

### 3.4. Reverse MR analysis

**Figure 4.**
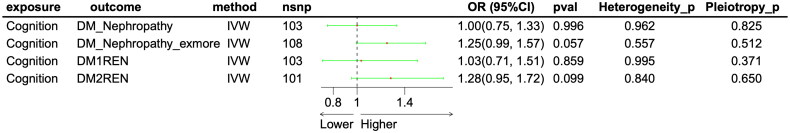
Forest plot displaying reverse Mendelian randomization results testing cognitive function’s effect on diabetic kidney disease risk. IVW: inverse variance weighted; nSNP: the number of SNPs used in the analysis; OR: odds ratio; pval: *p* value; heterogeneity *p: p* value of IVW heterogeneity test; pleiotropy *p: p* value of pleiotropy test.

Reverse MR analysis, with cognitive function as the exposure and DKD as the outcome, yielded non-significant causal estimates (IVW *p* > .05), the absence of reverse causality from cognitive function to DKD. sensitivity analyses, including heterogeneity test and MR-Egger intercept test for horizontal pleiotropy, confirmed the robustness of the findings, with no evidence of heterogeneity (*p* > .05) or pleiotropy (MR-Egger intercept *p* > .05) (Figure 4).

### 3.5. MVMR

**Figure 5.**
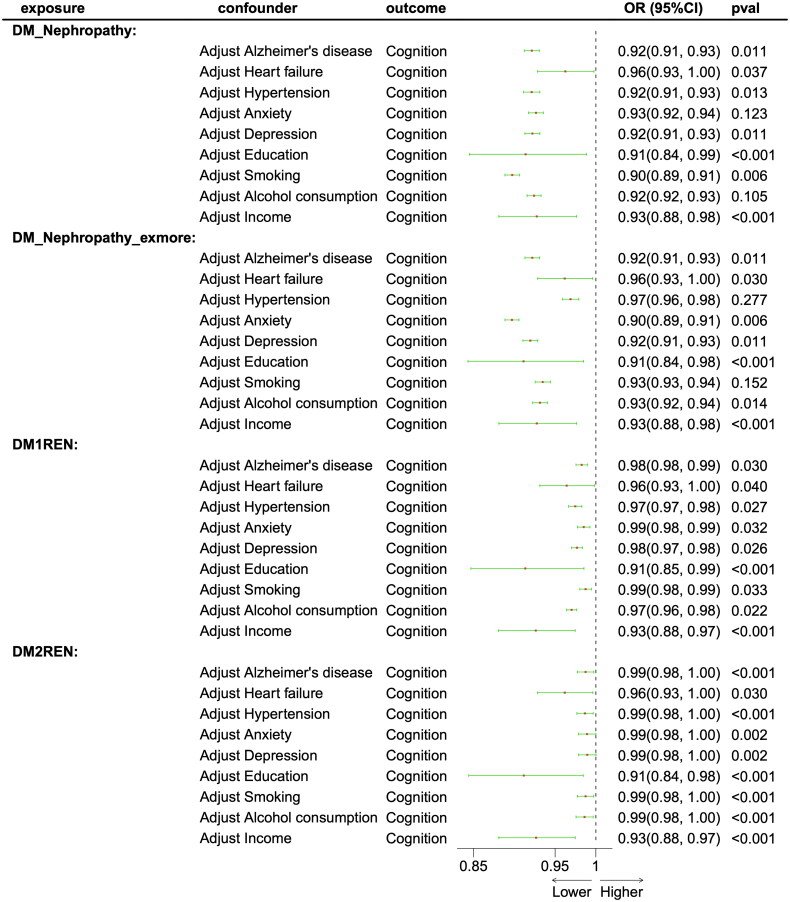
Forest plot displaying multivariate Mendelian randomization results examining the direct effect of diabetic kidney disease on cognitive function after adjustment for Alzheimer’s disease, heart failure, hypertension, anxiety, depression, education, smoking, alcohol consumption, and income. OR: odds ratio; 95% CI: 95% confidence interval; pval: *p* value.

MVMR analysis adjusted for nine potential confounders: Alzheimer’s disease, heart failure, hypertension, anxiety, depression, education, smoking, alcohol consumption, and income. Across all four DKD datasets, consistent and clinically meaningful effect sizes were observed for the DKD-CI association after adjustment for Alzheimer’s disease, heart failure, depression, education, and income. For example, following adjustment for Alzheimer’s disease, the causal association between DKD and CI was attenuated but remained statistically significant (adjusted OR = 0.92, 95% CI: 0.91–0.93 vs. unadjusted OR = 0.82, 95% CI: 0.78–0.85). However, some exceptions were observed; for instance, after adjusting for alcohol consumption, the association between DM nephropathy and cognitive function was no longer significant (*p* = .1049). Collectively, these results demonstrate that the vast majority of the observed causal relationship between DKD and CI persists after adjustment for potential confounders (Figure 5).

### 3.6. Genetic correlation analysis

LDSC analysis demonstrated significant SNP-based heritability for DKD across all datasets: DM nephropathy (*h*^2^ = 0.028), DM nephropathy exmore (*h*^2^ = 0.015), DM1REN (*h*^2^ = 0.012), and DM2REN (*h*^2^ = 0.009). The genetic correlation analyses demonstrated consistent and statistically significant positive associations between DKD and cognitive function in all four datasets (DM nephropathy: rg = 0.072; DM nephropathy exmore: rg = 0.168; DM1REN: rg = 0.109; DM2REN: rg = 0.201; Table 2). These robust genetic correlations (range: 0.072–0.201) provide compelling evidence for shared genetic architecture between DKD and cognitive function.

**Table 2.** Genetic correlation between diabetic kidney disease and cognitive function.

	*h* ^2^	Lambda GC	Mean Chi^2^	Intercept	Genetic correlation
DM nephropathy	0.028	1.077	1.082	0.964	0.072
DM nephropathy exmore	0.015	1.124	1.134	1.054	0.168
DM1REN	0.012	1.077	1.089	1.031	0.109
DM2REN	0.009	1.108	1.110	1.057	0.201

### 3.7. Colocalization and enrichment analysis

**Figure 6.**
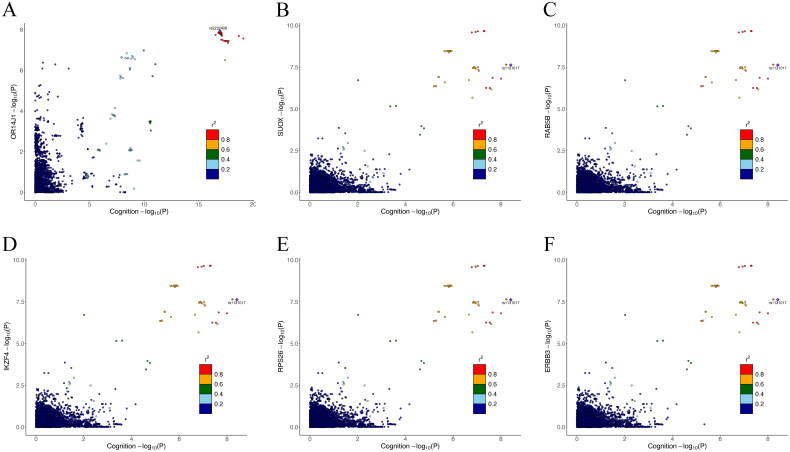
Genetic colocalization analysis between diabetic kidney disease and cognitive function at six susceptibility loci. Each panel displays: *X*-axis: −log10(*p* value) from cognitive function GWAS; *Y*-axis: −log10(*p* value) from diabetic kidney disease GWAS at the (A) OR14J1, (B) SUOX, (C) RAB5B, (D) IKZF4, (E) RPS26, and (F) ERBB3 loci; dots represent individual genetic variants colored by their linkage disequilibrium (*r*^2^) with the lead SNP. Analyses were performed using Coloc software with posterior probability >.90 defining significant colocalization.

Lead SNPs were identified using FUMA (Supplementary Table 7), and colocalization analysis was performed within a ±1 Mb region flanking these SNPs. Six genes – OR14J1, SUOX, RAB5B, IKZF4, RPS26, and ERBB3 – showed significant colocalization effects between DKD and cognitive function (Table 3 and Figure 6). To further explore their functional relevance, we analyzed the expression levels of these six genes in brain tissues and identified DEG sets for each gene. While no significant DEGs were detected for OR14J1, the remaining five genes (SUOX, RAB5B, IKZF4, RPS26, and ERBB3) showed substantial differential expression, with 4659, 6067, 5932, 2244, and 2991 DEGs identified, respectively. Gene Ontology enrichment analysis of these DEG sets revealed significant enrichment in key biological processes, including cytosolic ribosome, cytoplasmic translation, ribosomal subunit, structural constituent of ribosome, mitochondrial protein-containing complex, mitochondrial inner membrane, and oxidative phosphorylation. Additionally, KEGG pathway analysis demonstrated strong associations with neurodegenerative disorders (e.g., Parkinson’s disease, prion disease, amyotrophic lateral sclerosis, and Huntington’s disease) and metabolic pathways (e.g., oxidative phosphorylation and reactive oxygen species metabolism) (Supplementary Figure 3).

**Table 3.** Genes with colocalization effects between DKD and cognitive function.

PPH0	PPH1	PPH2	PPH3	PPH4	Gene
5.86E − 18	4.07E − 15	5.63E − 05	0.038153	0.961791	OR14J1
3.42E − 08	0.000188	1.12E − 05	0.06037	0.939431	SUOX
3.42E − 08	0.000188	1.12E − 05	0.06037	0.939431	RAB5B
3.42E − 08	0.000188	1.12E − 05	0.06037	0.939431	IKZF4
3.42E − 08	0.000188	1.12E − 05	0.060372	0.939429	RPS26
3.42E − 08	0.000188	1.12E − 05	0.060392	0.939409	ERBB3

## 4. Discussion

CI is a significant global health challenge, affecting an estimated over 55 million people worldwide, with many mild cases likely remaining undiagnosed [26]. Well-established risk factors for CI include obesity, diabetes, hypertension, physical inactivity, smoking, depression, and low educational attainment [27]. Diabetes mellitus and CI share numerous pathophysiological mechanisms, and population-based studies have shown an increased risk of CI in individuals with diabetes [28,29]. Similarly, patients with CKD are at a higher risk of CI. Studies have demonstrated that reduced estimated glomerular filtration rate (eGFR) is associated with cognitive decline, with each 10 mL/min/1.73 m^2^ increase in eGFR linked to a 4.8% lower risk of CI [30]. According to the National Health and Nutrition Examination Survey (NHANES), patients with advanced CKD (stages G4 and G5) and low eGFR exhibit poorer overall cognitive function compared to those without CKD [31]. In addition to eGFR, several other factors contribute to CI in CKD patients. Hypertension and heart failure, common comorbidities in diabetes and CKD, are associated with an elevated risk of CI, particularly in patients with an eGFR >45 mL/min/1.73 m^2^ [32,33]. Furthermore, CKD patients often experience anxiety and depression due to the demands of long-term treatment, medication, and dietary restrictions. A prospective cohort study in the UK found that anxiety and depression are significant risk factors for CI in CKD patients [34]. Additionally, heavy alcohol consumption in CKD patients is associated with a higher risk of CI compared to light drinkers [35]. Given these findings, hypertension, heart failure, alcohol consumption, anxiety, and depression were included as confounders in this study.

CI significantly impacts the prognosis of patients DKD, leading to reduced treatment adherence, diminished quality of life, and increased mortality risk. However, therapeutic options for DKD patients with CI are limited, and prevention remains the primary strategy. Therefore, understanding the relationship between DKD and CI is crucial for identifying potential therapeutic targets and improving patient outcomes.

Several mechanisms have been proposed to explain the development of CI in DKD patients. First, prolonged exposure of the brain and kidneys to high-flow, low-resistance environments can lead to capillary basement membrane thickening, lumen stenosis, increased microcirculation pressure, elevated vascular permeability, and ultimately, organ damage [36]. A similar microangiopathic process occurs in both the kidneys and brains of diabetic patients [37]. A cross-sectional study of 3011 patients with T2DM found that microangiopathy significantly affects cognitive function, particularly memory and executive speed [38]. Second, inflammation, oxidative stress, and endothelial injury play critical roles in the development of both DKD and CI [39]. DKD patients often exhibit a chronic inflammatory state, and inflammatory mediators are closely linked to brain inflammation, which is considered a potential mechanism for CI [40]. Endothelial damage can impair the blood–brain barrier, allowing inflammatory mediators and toxic substances to infiltrate the brain, leading to cognitive decline. Third, abnormal deposition of β-amyloid protein (Aβ) is a key mechanism underlying cognitive dysfunction [41,42]. Neurotoxic Aβ can directly impair synaptic function and activate microglia and astrocytes, resulting in blood–brain barrier disruption and microcirculation disturbances. Clinical studies have shown elevated Aβ levels in patients with type 2 diabetes, potentially due to hyperinsulinemia, which reduces Aβ degradation and increases its deposition, contributing to cognitive decline [42]. Since Aβ clearance is partially dependent on kidney function, impaired renal function in DKD patients may exacerbate cognitive dysfunction by reducing Aβ clearance [43]. Additionally, reduced clearance of uremic toxins and hemodynamic changes during dialysis may further contribute to CI in DKD patients [44].

Despite these insights, the relationship between DKD and CI remains incompletely understood, and the potential shared genetic susceptibility or causal relationship between the two conditions has yet to be fully elucidated.

This two-sample MR analysis provides the first genetic evidence supporting a causal relationship between DKD and CI. The MR analysis demonstrated a consistent causal effect of DKD on CI across multiple datasets, with ORs indicating reduced cognitive function in DKD patients. This finding aligns with previous observational studies suggesting that DKD is a risk factor for CI [3–5]. The non-significant reverse MR results strengthen our interpretation by suggesting that while DKD appears to contribute to CI through genetically mediated mechanisms, we did not detect significant genetic evidence for cognition influencing DKD risk. Moreover, the robustness of our results was supported by sensitivity analyses, which showed no significant heterogeneity or horizontal pleiotropy.

Our MVMR analysis established a significant causal effect of DKD on CI following comprehensive adjustment for multiple confounder categories: comorbidities (Alzheimer’s disease, heart failure, hypertension, anxiety, depression), education level, socioeconomic status (income), and lifestyle factors (smoking and alcohol consumption). While we observed changes in effect sizes after adjusting for specific confounders (such as alcohol consumption), the consistent direction of effects across analyses continues to support a causal relationship. These results highlight both the importance of comprehensive confounding adjustment in genetic studies and the complex biological and lifestyle interactions influencing cognitive decline in DKD patients. The observed variations in association strength likely reflect the multifactorial nature of this relationship, involving both direct pathological mechanisms and indirect pathways mediated by lifestyle factors. Notably, while shared risk factors partially mediate this association, the persistent independent effect suggests DKD contributes to CI through distinct biological mechanisms.

The genetic correlation analysis using LDSC revealed a significant positive correlation between DKD and CI (rg range: 0.072–0.201), indicating a shared genetic basis between these conditions. This consistent directional relationship across all analyzed datasets suggests common pathogenic mechanisms that warrant further investigation.

Colocalization analysis identified six candidate genes (OR14J1, SUOX, RAB5B, IKZF4, RPS26, and ERBB3) potentially shared by DKD and CI, several of which have established associations with these conditions. ERBB3 encodes a receptor tyrosine kinase. In the central nervous system, ERBB signaling pathway is essential for neuronal development and maintenance. ERBB3 dysregulation is linked to neurodegenerative conditions and may contribute to CI by regulating neuronal survival pathways [45]. In renal pathology, ERBB3 activation drives mesangial cell proliferation and fibrosis, directly promoting DKD progression [46]. Similarly, the RAB5B gene product is a small GTPase belonging to the Ras/Rab superfamily. Accumulating evidence has established the critical involvement of Rab proteins in neurodegenerative processes and CI [47,48], with emerging research suggesting Rab5’s potential role in axonal regeneration [49]. Dysregulation of Rab5-mediated pathways may also contribute to the accelerated aging phenotype characteristic of DKD [50]. Notably, recent clinical trials have demonstrated that certain glucose-lowering drugs, including GLP-1 receptor agonists and SGLT2 inhibitors, exhibit both nephroprotective effects and potential cognitive benefits [51–53]. Interestingly, our identified genetic variants in sulfate metabolism genes (SUOX), which may regulate oxidative stress pathways – a known target of SGLT2 inhibitors – might offer new perspectives on CI in DKD patients. These findings warrant further investigation into the mechanistic links between genetic factors and drug efficacy. Pathway enrichment analysis further demonstrated that these genes are significantly enriched in key biological processes, including cytosolic ribosome assembly, cytoplasmic translation, and mitochondrial oxidative phosphorylation. KEGG analysis further linked these genes to neurological disorders, as well as pathways involving reactive oxygen species and mitochondrial dysfunction. These findings suggest that oxidative stress, mitochondrial dysfunction, and impaired protein homeostasis may serve as shared mechanisms linking DKD to CI. These insights not only deepen our understanding of the shared pathophysiology between DKD and CI but also point to potential therapeutic targets. In patients with diabetes, interventions targeting oxidative stress or mitochondrial function could benefit both renal and cognitive health.

Based on our study findings, we recommend that patients with DKD undergo standardized cognitive monitoring, including annual screening using validated tools (MMSE/MoCA). Particular attention should be given to DKD patients harboring risk variants in identified genes, as they may represent a genetically defined subgroup warranting prioritized cognitive surveillance. When clinically indicated, cerebrospinal fluid biomarker analysis (Aβ42, Aβ42/Aβ40, total tau, and p-tau181) [54] and brain MRI should be considered. We further suggest implementing personalized treatment strategies that prioritize the use of potentially neuroprotective glucose-lowering agents (e.g., SGLT2 inhibitors, GLP-1RAs) for high-risk patients and establishing multidisciplinary care teams (endocrinology/nephrology/neurology). Future research should focus on integrating genetic risk assessment into routine diabetes management and developing targeted interventions.

Our study has several strengths, including the use of large-scale GWAS data, rigorous MR methods, and comprehensive sensitivity analyses. However, some limitations should be acknowledged. First, as the GWAS data were predominantly derived from European-ancestry populations, the generalizability of our findings to other ethnic groups remains uncertain due to potential differences in genetic architecture and environmental exposures, necessitating validation in diverse cohorts. Second, while our integrated genomic analyses have identified several promising candidate genes linking DKD and CI, we recognize the importance of systematic experimental validation to fully establish their biological roles. Future studies employing cellular and animal models will be essential to confirm these genes’ involvement in the kidney–brain axis and explore their therapeutic potential. Finally, the potential influence of unmeasured confounders cannot be entirely ruled out. In general, the interpretation of these results should be approached with caution, and further large-scale clinical and epidemiological studies are needed to validate these findings.

## 5. Conclusions

In conclusion, our study provides strong evidence for a causal relationship between DKD and CI, supported by genetic correlations and shared biological pathways. The identification of colocalized genes offers new insights into the molecular mechanisms linking DKD to CI. These findings highlight the importance of early detection and management of DKD to mitigate the risk of cognitive decline. Future studies should validate candidate genes in experimental models, develop genetic risk scores for clinical prediction, and test targeted therapies. These findings should inform integrated diabetes care protocols.

## Supplementary Material

Supplementary Figure 1.tiff

Supplementary Table 1.docx

Supplementary Table 7.docx

Supplementary Table 2.docx

Supplementary Figure 3.tiff

Supplementary Table 5.docx

Supplementary Table 6.docx

Supplementary Table 4.docx

Supplementary Table 3.docx

Supplementary Figure 2.tiff

## Data Availability

All data generated during the study can be obtained upon reasonable request from the corresponding author.
