# Supplementary figures and images for: Causal relationship and shared genetic pathways between diabetic kidney disease and cognitive impairment: a Mendelian randomization study
Source: Ren Fail. 2025 Jul 1;47(1):2525471. doi: 10.1080/0886022X.2025.2525471 (PMC12217110; doi:10.1080/0886022X.2025.2525471)

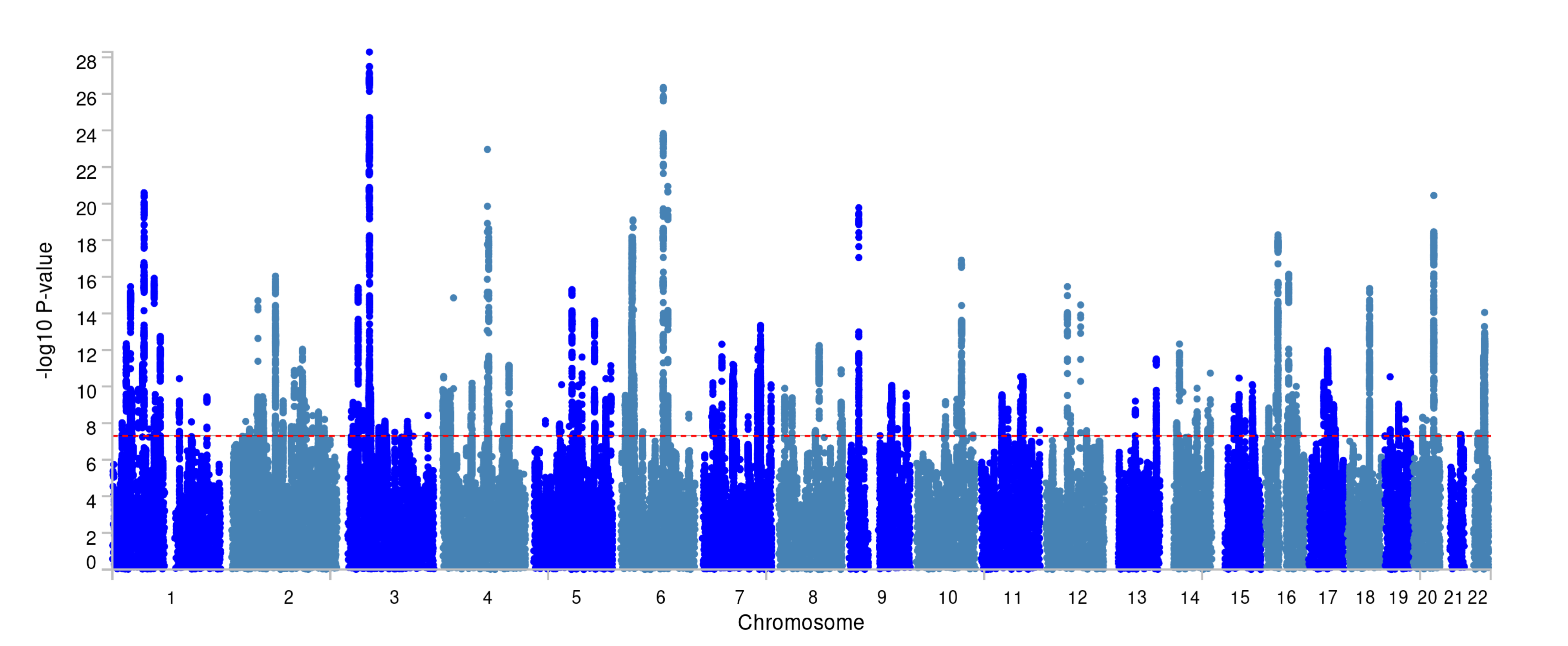

Supplement: Supplementary Figure 1.tiff [file IRNF_A_2525471_SM3601.tiff]

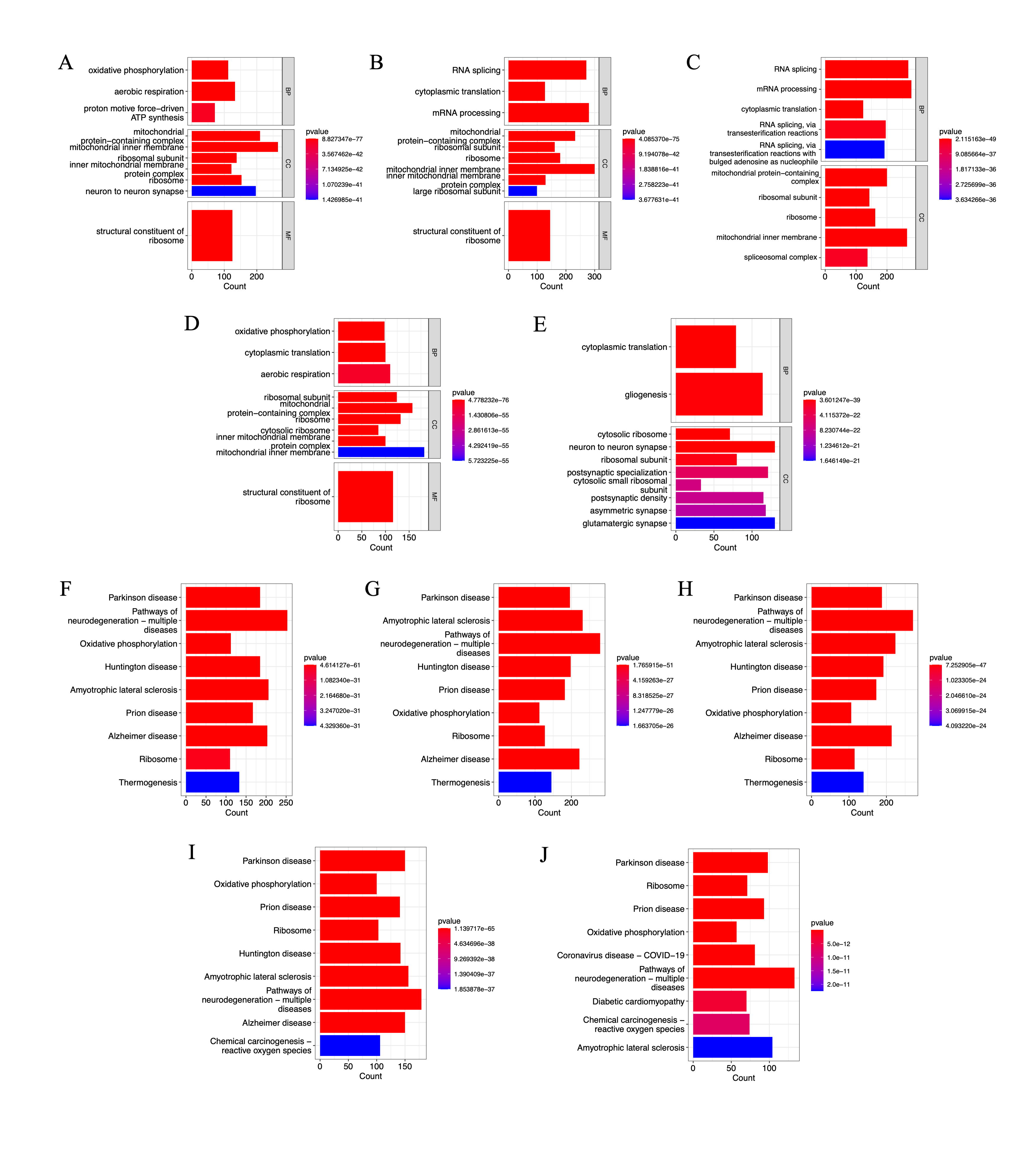

Supplement: Supplementary Figure 3.tiff [file IRNF_A_2525471_SM3597.tiff]

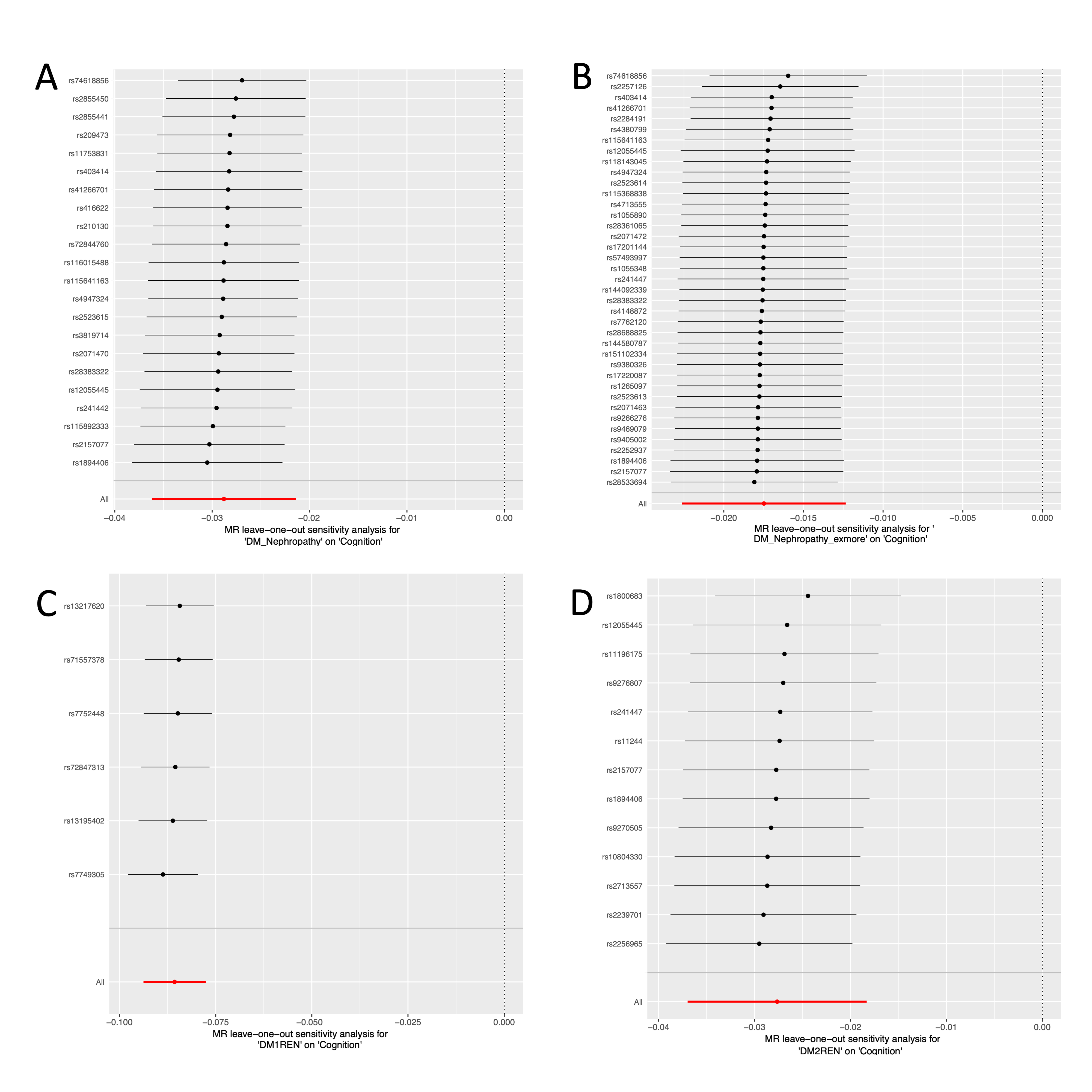

Supplement: Supplementary Figure 2.tiff [file IRNF_A_2525471_SM3592.tiff]
