# Supplementary material for: Causal relationship and shared genetic pathways between diabetic kidney disease and cognitive impairment: a Mendelian randomization study
Source: Ren Fail. 2025 Jul 1;47(1):2525471. doi: 10.1080/0886022X.2025.2525471 (PMC12217110; doi:10.1080/0886022X.2025.2525471)
Supplement: Supplementary Table 1.docx [file IRNF_A_2525471_SM3600.docx]

**Supplementary Table 1. Characteristics of the exposure datasets used in the Mendelian randomization analysis**

| **Dataset** | **Sample size** | **Population** | **Date source** |
| --- | --- | --- | --- |
| DM Nephropathy | 84,386 | European | FinnGen |
| DM Nephropathy exmore | 312,650 | European | FinnGen |
| DM1REN | 309,859 | European | FinnGen |
| DM2REN | 309,859 | European | FinnGen |

DM Nephropathy, diabetic nephropathy; DM Nephropathy exmore, diabetic nephropathy (more control exclusions); DM1REN, Type 1 diabetes with renal complication; DM2REN, Type 2 diabetes with renal complication
