# Supplementary material for: Causal relationship and shared genetic pathways between diabetic kidney disease and cognitive impairment: a Mendelian randomization study
Source: Ren Fail. 2025 Jul 1;47(1):2525471. doi: 10.1080/0886022X.2025.2525471 (PMC12217110; doi:10.1080/0886022X.2025.2525471)
Supplement: Supplementary Table 7.docx [file IRNF_A_2525471_SM3599.docx]

**Supplementary Table 7. Lead SNPs significantly associated with cognitive function identified through FUMA analysis (P < 5 × 10⁻⁸)**

| **uniqID** | **LeadSNP** | **chr** | **pos** | **p** | **nIndSigSNPs** |
| --- | --- | --- | --- | --- | --- |
| 1:22430069:A:G | rs2143103 | 1 | 22430069 | 9.64E-09 | 1 |
| 1:32106494:A:G | rs7546297 | 1 | 32106494 | 4.55E-13 | 3 |
| 1:37865954:A:G | rs875361 | 1 | 37865954 | 1.50E-08 | 2 |
| 1:41781151:C:T | rs1892419 | 1 | 41781151 | 3.47E-16 | 5 |
| 1:41833089:G:T | rs2364543 | 1 | 41833089 | 6.71E-15 | 2 |
| 1:43950265:C:G | rs2782653 | 1 | 43950265 | 4.50E-10 | 4 |
| 1:44405563:C:T | rs11210937 | 1 | 44405563 | 2.06E-10 | 3 |
| 1:59560337:C:T | rs1831539 | 1 | 59560337 | 1.43E-10 | 1 |
| 1:69427576:A:C | rs9436866 | 1 | 69427576 | 6.01E-12 | 3 |
| 1:72588119:A:G | rs12128707 | 1 | 72588119 | 9.03E-21 | 8 |
| 1:72634912:A:G | rs1157072 | 1 | 72634912 | 4.70E-09 | 3 |
| 1:72749848:C:T | rs3128341 | 1 | 72749848 | 2.52E-21 | 9 |
| 1:84417585:C:T | rs41293013 | 1 | 84417585 | 2.11E-08 | 1 |
| 1:96175936:A:T | rs10874938 | 1 | 96175936 | 1.19E-16 | 2 |
| 1:98288581:C:T | rs78164635 | 1 | 98288581 | 2.75E-10 | 1 |
| 1:98588885:C:T | rs1473474 | 1 | 98588885 | 4.44E-09 | 1 |
| 1:103586052:C:T | rs6577356 | 1 | 1.04E+08 | 3.56E-09 | 2 |
| 1:110030945:A:G | rs1144593 | 1 | 1.10E+08 | 1.82E-13 | 5 |
| 1:153797015:A:G | rs112780312 | 1 | 1.54E+08 | 3.73E-11 | 1 |
| 1:181535715:A:G | rs78382112 | 1 | 1.82E+08 | 8.54E-09 | 1 |
| 1:216830128:A:T | rs11117646 | 1 | 2.17E+08 | 3.66E-10 | 3 |
| 2:32736043:C:T | rs17428810 | 2 | 32736043 | 8.10E-09 | 1 |
| 2:41606931:A:G | rs6708515 | 2 | 41606931 | 2.11E-08 | 1 |
| 2:44185129:C:T | rs4347883 | 2 | 44185129 | 2.79E-08 | 1 |
| 2:57923006:G:T | rs13012916 | 2 | 57923006 | 2.77E-08 | 1 |
| 2:57948003:C:T | rs17049085 | 2 | 57948003 | 3.44E-09 | 1 |
| 2:60713235:A:G | rs10189857 | 2 | 60713235 | 2.03E-15 | 8 |
| 2:71534617:A:T | rs4470366 | 2 | 71534617 | 3.61E-10 | 2 |
| 2:73387386:C:G | rs6760964 | 2 | 73387386 | 5.73E-10 | 4 |
| 2:73837751:C:T | rs6546856 | 2 | 73837751 | 1.86E-08 | 2 |
| 2:100608840:C:T | rs34795510 | 2 | 1.01E+08 | 2.21E-10 | 4 |
| 2:100871186:A:G | rs11123820 | 2 | 1.01E+08 | 9.17E-17 | 7 |
| 2:101322193:C:G | rs2970991 | 2 | 1.01E+08 | 2.11E-08 | 1 |
| 2:117697406:C:G | rs6718450 | 2 | 1.18E+08 | 9.53E-09 | 1 |
| 2:118627164:G:T | rs78358737 | 2 | 1.19E+08 | 6.18E-10 | 2 |
| 2:137512404:C:T | rs72850040 | 2 | 1.38E+08 | 1.56E-08 | 1 |
| 2:144250487:C:T | rs13428598 | 2 | 1.44E+08 | 1.32E-11 | 2 |
| 2:145078775:C:T | rs62169190 | 2 | 1.45E+08 | 2.74E-08 | 1 |
| 2:155731956:A:G | rs2652430 | 2 | 1.56E+08 | 7.38E-09 | 1 |
| 2:156911600:G:T | rs72906064 | 2 | 1.57E+08 | 2.09E-08 | 1 |
| 2:157487273:C:G | rs13425585 | 2 | 1.57E+08 | 1.11E-11 | 4 |
| 2:161385291:C:T | rs7419274 | 2 | 1.61E+08 | 4.89E-08 | 1 |
| 2:162101261:A:G | rs11678980 | 2 | 1.62E+08 | 1.43E-10 | 2 |
| 2:162802184:A:T | rs11693702 | 2 | 1.63E+08 | 9.17E-13 | 3 |
| 2:163976035:C:G | rs7588384 | 2 | 1.64E+08 | 4.26E-09 | 2 |
| 2:166672949:C:T | rs13413443 | 2 | 1.67E+08 | 5.56E-10 | 2 |
| 2:171671166:C:T | rs872123 | 2 | 1.72E+08 | 1.39E-09 | 1 |
| 2:186243561:A:C | rs7599860 | 2 | 1.86E+08 | 3.92E-09 | 1 |
| 2:198929896:C:G | rs7573001 | 2 | 1.99E+08 | 2.35E-08 | 1 |
| 2:199553600:A:G | rs114125694 | 2 | 2.00E+08 | 2.48E-09 | 1 |
| 2:212590505:C:T | rs7556896 | 2 | 2.13E+08 | 1.88E-08 | 1 |
| 2:213403091:A:G | rs73989053 | 2 | 2.13E+08 | 6.31E-09 | 1 |
| 3:8246179:C:T | rs12635303 | 3 | 8246179 | 2.21E-09 | 1 |
| 3:12475088:C:T | rs7626560 | 3 | 12475088 | 7.34E-10 | 3 |
| 3:24062661:A:G | rs6550835 | 3 | 24062661 | 3.89E-16 | 7 |
| 3:35527409:C:T | rs1523048 | 3 | 35527409 | 2.06E-09 | 1 |
| 3:48584813:C:T | rs73078305 | 3 | 48584813 | 3.30E-09 | 3 |
| 3:49314960:A:G | rs56306491 | 3 | 49314960 | 4.38E-11 | 7 |
| 3:49890613:C:T | rs2352974 | 3 | 49890613 | 5.19E-29 | 18 |
| 3:52556890:A:G | rs4434138 | 3 | 52556890 | 1.27E-10 | 6 |
| 3:71545170:A:C | rs11720523 | 3 | 71545170 | 1.73E-08 | 2 |
| 3:85327518:A:G | rs73845427 | 3 | 85327518 | 7.69E-09 | 4 |
| 3:85941811:A:G | rs111246959 | 3 | 85941811 | 2.23E-08 | 1 |
| 3:108103433:C:T | rs3860537 | 3 | 1.08E+08 | 3.14E-08 | 1 |
| 3:135953220:A:G | rs9826454 | 3 | 1.36E+08 | 4.17E-08 | 1 |
| 3:137057630:A:C | rs66752974 | 3 | 1.37E+08 | 7.92E-09 | 2 |
| 3:139703395:G:T | rs34903090 | 3 | 1.40E+08 | 2.93E-08 | 1 |
| 3:184069126:C:G | rs73189617 | 3 | 1.84E+08 | 3.87E-09 | 1 |
| 4:696848:C:T | rs12646225 | 4 | 696848 | 1.48E-10 | 2 |
| 4:2717690:C:T | rs2295499 | 4 | 2717690 | 2.85E-11 | 3 |
| 4:10704137:A:T | rs13120565 | 4 | 10704137 | 5.66E-10 | 1 |
| 4:17853921:A:G | rs16895888 | 4 | 17853921 | 1.80E-10 | 2 |
| 4:25408838:A:G | rs34811474 | 4 | 25408838 | 1.42E-15 | 1 |
| 4:65831184:C:T | rs3943667 | 4 | 65831184 | 3.56E-09 | 1 |
| 4:67970101:A:G | rs6819372 | 4 | 67970101 | 6.57E-11 | 1 |
| 4:103188709:C:T | rs13107325 | 4 | 1.03E+08 | 1.09E-23 | 12 |
| 4:103190828:C:G | rs4699006 | 4 | 1.03E+08 | 1.37E-10 | 7 |
| 4:106217358:G:T | rs2726513 | 4 | 1.06E+08 | 2.37E-19 | 6 |
| 4:140780768:A:T | rs35853157 | 4 | 1.41E+08 | 1.48E-08 | 1 |
| 4:152592933:A:G | rs6535809 | 4 | 1.53E+08 | 6.90E-12 | 2 |
| 5:26880925:A:G | rs75973558 | 5 | 26880925 | 7.38E-09 | 1 |
| 5:59636727:C:T | rs6860626 | 5 | 59636727 | 1.11E-08 | 1 |
| 5:62991802:G:T | rs1812587 | 5 | 62991802 | 2.84E-08 | 1 |
| 5:64020316:G:T | rs80170948 | 5 | 64020316 | 7.99E-11 | 1 |
| 5:81612479:A:G | rs658912 | 5 | 81612479 | 1.50E-08 | 1 |
| 5:87943710:A:C | rs13163336 | 5 | 87943710 | 5.04E-16 | 6 |
| 5:88029208:C:T | rs41352752 | 5 | 88029208 | 1.18E-08 | 3 |
| 5:88170066:C:T | rs304136 | 5 | 88170066 | 1.63E-10 | 4 |
| 5:91096263:A:T | rs34156429 | 5 | 91096263 | 3.65E-09 | 2 |
| 5:92477890:A:C | rs4342312 | 5 | 92477890 | 1.05E-09 | 1 |
| 5:101105998:C:T | rs1479073 | 5 | 1.01E+08 | 1.92E-08 | 1 |
| 5:102726073:C:T | rs74944275 | 5 | 1.03E+08 | 8.47E-11 | 3 |
| 5:111010072:C:T | rs26046 | 5 | 1.11E+08 | 2.44E-12 | 3 |
| 5:139545748:A:G | rs4463213 | 5 | 1.40E+08 | 2.56E-14 | 6 |
| 5:140054485:C:T | rs2530241 | 5 | 1.40E+08 | 1.03E-08 | 4 |
| 5:165473194:A:G | rs830383 | 5 | 1.65E+08 | 3.73E-11 | 5 |
| 5:176908731:A:C | rs335426 | 5 | 1.77E+08 | 7.24E-12 | 2 |
| 6:11508671:A:C | rs620729 | 6 | 11508671 | 3.05E-10 | 2 |
| 6:21956404:A:G | rs6903716 | 6 | 21956404 | 4.62E-09 | 1 |
| 6:25480328:G:T | rs34991172 | 6 | 25480328 | 1.31E-10 | 10 |
| 6:26374658:A:G | rs2073526 | 6 | 26374658 | 9.52E-11 | 14 |
| 6:26599509:A:G | rs45527431 | 6 | 26599509 | 6.67E-19 | 19 |
| 6:27698837:C:T | rs13202295 | 6 | 27698837 | 1.70E-18 | 16 |
| 6:28934352:C:T | rs148696809 | 6 | 28934352 | 7.79E-20 | 6 |
| 6:50790633:A:G | rs6930924 | 6 | 50790633 | 3.99E-08 | 1 |
| 6:51690108:G:T | rs56135595 | 6 | 51690108 | 2.97E-08 | 1 |
| 6:98337903:A:G | rs6928545 | 6 | 98337903 | 2.97E-10 | 7 |
| 6:98550289:A:C | rs1906252 | 6 | 98550289 | 4.44E-27 | 10 |
| 6:99292136:A:G | rs2294405 | 6 | 99292136 | 1.64E-08 | 3 |
| 6:99532265:A:G | rs9385616 | 6 | 99532265 | 2.31E-09 | 1 |
| 6:108864419:C:T | rs9384679 | 6 | 1.09E+08 | 1.14E-21 | 9 |
| 6:157140077:A:T | rs287883 | 6 | 1.57E+08 | 3.28E-09 | 1 |
| 7:21467190:C:T | rs39302 | 7 | 21467190 | 4.49E-09 | 1 |
| 7:24171927:C:T | rs6952104 | 7 | 24171927 | 6.32E-11 | 2 |
| 7:32334880:C:T | rs215603 | 7 | 32334880 | 1.44E-08 | 1 |
| 7:32493169:A:T | rs1580019 | 7 | 32493169 | 2.49E-09 | 1 |
| 7:44800176:G:T | rs3735478 | 7 | 44800176 | 4.85E-13 | 4 |
| 7:68887851:C:T | rs10225320 | 7 | 68887851 | 1.32E-10 | 3 |
| 7:69607509:C:G | rs117463770 | 7 | 69607509 | 3.04E-11 | 5 |
| 7:69735251:A:G | rs12112638 | 7 | 69735251 | 2.31E-11 | 8 |
| 7:69993342:C:G | rs3113262 | 7 | 69993342 | 6.18E-12 | 5 |
| 7:71741232:C:G | rs35526560 | 7 | 71741232 | 6.71E-12 | 2 |
| 7:105063372:C:G | rs12535854 | 7 | 1.05E+08 | 4.46E-09 | 1 |
| 7:127082497:A:G | rs4731365 | 7 | 1.27E+08 | 3.20E-10 | 5 |
| 7:127733822:A:C | rs17151739 | 7 | 1.28E+08 | 8.54E-13 | 6 |
| 7:128410012:A:G | rs1043595 | 7 | 1.28E+08 | 3.68E-11 | 1 |
| 7:133531432:C:T | rs6975134 | 7 | 1.34E+08 | 4.55E-14 | 14 |
| 7:157536448:G:T | rs1009950 | 7 | 1.58E+08 | 8.15E-11 | 3 |
| 8:14002020:G:T | rs13253386 | 8 | 14002020 | 1.26E-10 | 2 |
| 8:20915316:A:G | rs1473634 | 8 | 20915316 | 9.39E-10 | 1 |
| 8:31013470:A:G | rs2737339 | 8 | 31013470 | 4.14E-10 | 3 |
| 8:84490118:A:G | rs34531385 | 8 | 84490118 | 2.55E-08 | 1 |
| 8:93164505:A:T | rs991871 | 8 | 93164505 | 5.72E-13 | 2 |
| 8:141545193:C:T | rs2977464 | 8 | 1.42E+08 | 9.86E-09 | 1 |
| 8:143311653:A:G | rs4976976 | 8 | 1.43E+08 | 1.24E-11 | 2 |
| 8:145744429:C:T | rs2721173 | 8 | 1.46E+08 | 6.13E-09 | 1 |
| 9:23362311:G:T | rs11793831 | 9 | 23362311 | 1.69E-20 | 7 |
| 9:23805569:C:T | rs702222 | 9 | 23805569 | 1.66E-11 | 1 |
| 9:72110562:C:T | rs11138947 | 9 | 72110562 | 4.83E-08 | 1 |
| 9:96271752:A:G | rs4744250 | 9 | 96271752 | 9.34E-10 | 3 |
| 9:98270168:A:G | rs1355620 | 9 | 98270168 | 1.24E-08 | 1 |
| 9:99100713:C:T | rs10990610 | 9 | 99100713 | 8.69E-11 | 2 |
| 9:111875463:A:G | rs2439649 | 9 | 1.12E+08 | 1.98E-08 | 1 |
| 9:131942676:A:G | rs2005078 | 9 | 1.32E+08 | 2.35E-10 | 1 |
| 9:134866271:C:T | rs7044246 | 9 | 1.35E+08 | 1.96E-08 | 1 |
| 10:65016721:A:G | rs7896910 | 10 | 65016721 | 2.38E-08 | 1 |
| 10:67188211:A:T | rs12773747 | 10 | 67188211 | 6.73E-10 | 1 |
| 10:93349867:G:T | rs72821233 | 10 | 93349867 | 4.88E-10 | 4 |
| 10:101912194:C:T | rs1408579 | 10 | 1.02E+08 | 4.11E-09 | 1 |
| 10:103565960:C:G | rs3740422 | 10 | 1.04E+08 | 1.23E-17 | 9 |
| 10:104082688:G:T | rs12772375 | 10 | 1.04E+08 | 2.38E-10 | 6 |
| 10:106996964:C:T | rs7895991 | 10 | 1.07E+08 | 2.67E-08 | 1 |
| 10:130113462:G:T | rs2799399 | 10 | 1.30E+08 | 4.55E-08 | 1 |
| 11:47441664:C:T | rs2242511 | 11 | 47441664 | 4.10E-08 | 3 |
| 11:47640429:C:G | rs1064608 | 11 | 47640429 | 2.86E-10 | 3 |
| 11:61313525:C:T | rs77128898 | 11 | 61313525 | 1.72E-09 | 2 |
| 11:90513272:C:G | rs35293660 | 11 | 90513272 | 2.94E-11 | 4 |
| 11:95561682:A:G | rs602512 | 11 | 95561682 | 2.92E-11 | 2 |
| 11:133814713:A:G | rs4937860 | 11 | 1.34E+08 | 2.40E-08 | 1 |
| 12:49422094:A:G | rs10875914 | 12 | 49422094 | 3.45E-16 | 3 |
| 12:49661715:C:T | rs11168951 | 12 | 49661715 | 4.35E-08 | 1 |
| 12:53605344:C:T | rs3741434 | 12 | 53605344 | 4.68E-08 | 1 |
| 12:56435929:C:G | rs1131017 | 12 | 56435929 | 3.85E-09 | 1 |
| 12:79685226:C:T | rs7963801 | 12 | 79685226 | 3.46E-15 | 2 |
| 12:93327911:A:T | rs56290130 | 12 | 93327911 | 2.59E-08 | 2 |
| 13:58548511:C:G | rs3843954 | 13 | 58548511 | 6.33E-10 | 2 |
| 13:106590549:A:C | rs7999524 | 13 | 1.07E+08 | 1.27E-09 | 4 |
| 13:106640324:A:G | rs2478281 | 13 | 1.07E+08 | 3.10E-12 | 7 |
| 14:26935221:A:G | rs1245214 | 14 | 26935221 | 9.58E-09 | 2 |
| 14:30072382:C:T | rs1108732 | 14 | 30072382 | 4.21E-08 | 1 |
| 14:33292743:A:C | rs2239647 | 14 | 33292743 | 4.71E-13 | 4 |
| 14:41134168:A:G | rs35760956 | 14 | 41134168 | 2.02E-10 | 1 |
| 14:69708241:A:G | rs1043254 | 14 | 69708241 | 6.61E-09 | 1 |
| 14:73529094:A:G | rs2806048 | 14 | 73529094 | 1.25E-10 | 2 |
| 14:98546911:C:T | rs17698580 | 14 | 98546911 | 2.03E-09 | 2 |
| 14:104018455:A:G | rs10129426 | 14 | 1.04E+08 | 1.87E-11 | 2 |
| 15:40705417:A:G | rs2289328 | 15 | 40705417 | 5.71E-09 | 1 |
| 15:41222487:C:G | rs12441495 | 15 | 41222487 | 1.24E-09 | 3 |
| 15:51965186:C:T | rs11637716 | 15 | 51965186 | 2.16E-09 | 3 |
| 15:52196830:C:T | rs1035738 | 15 | 52196830 | 3.47E-11 | 3 |
| 15:65738080:C:T | rs72739469 | 15 | 65738080 | 7.51E-09 | 1 |
| 15:82546946:G:T | rs12439619 | 15 | 82546946 | 7.94E-11 | 4 |
| 15:84361518:A:G | rs11259916 | 15 | 84361518 | 3.53E-08 | 1 |
| 16:7253286:C:G | rs12325273 | 16 | 7253286 | 3.53E-08 | 1 |
| 16:7657373:A:G | rs1507010 | 16 | 7657373 | 1.52E-09 | 2 |
| 16:13156649:A:G | rs276626 | 16 | 13156649 | 4.81E-08 | 1 |
| 16:24803620:A:G | rs1862451 | 16 | 24803620 | 2.81E-10 | 2 |
| 16:28871191:C:G | rs12448902 | 16 | 28871191 | 5.12E-19 | 13 |
| 16:50951373:C:T | rs4785480 | 16 | 50951373 | 3.44E-09 | 1 |
| 16:51577196:C:T | rs2647995 | 16 | 51577196 | 4.99E-09 | 1 |
| 16:53498655:C:G | rs8054299 | 16 | 53498655 | 7.22E-17 | 4 |
| 16:62075138:A:G | rs12446238 | 16 | 62075138 | 2.77E-10 | 1 |
| 16:70756181:G:T | rs62047970 | 16 | 70756181 | 9.90E-11 | 3 |
| 16:71579667:A:C | rs310333 | 16 | 71579667 | 2.74E-08 | 1 |
| 16:71993386:A:T | rs4788584 | 16 | 71993386 | 5.45E-09 | 1 |
| 16:76524013:C:G | rs8058881 | 16 | 76524013 | 4.14E-08 | 1 |
| 17:34952789:C:T | rs35258073 | 17 | 34952789 | 5.76E-11 | 1 |
| 17:43383100:A:G | rs9908330 | 17 | 43383100 | 4.48E-08 | 3 |
| 17:43569909:C:T | rs146746174 | 17 | 43569909 | 1.08E-12 | 5 |
| 17:43781083:C:T | rs117560908 | 17 | 43781083 | 2.11E-10 | 4 |
| 17:44800046:A:G | rs7224296 | 17 | 44800046 | 6.06E-10 | 6 |
| 17:47090785:C:T | rs11079849 | 17 | 47090785 | 5.96E-11 | 1 |
| 17:57095212:C:T | rs74370218 | 17 | 57095212 | 9.81E-10 | 1 |
| 17:61052949:C:T | rs72843145 | 17 | 61052949 | 2.13E-08 | 1 |
| 18:50907365:C:T | rs11662271 | 18 | 50907365 | 4.39E-16 | 7 |
| 19:713148:C:T | rs34802460 | 19 | 713148 | 4.94E-08 | 1 |
| 19:12530177:A:G | rs17002025 | 19 | 12530177 | 2.96E-11 | 2 |
| 19:13113641:C:T | rs10411958 | 19 | 13113641 | 2.29E-08 | 2 |
| 19:31866978:C:T | rs116931822 | 19 | 31866978 | 4.46E-09 | 1 |
| 19:31952890:A:G | rs7256776 | 19 | 31952890 | 9.28E-10 | 2 |
| 19:47548678:A:G | rs889169 | 19 | 47548678 | 6.13E-09 | 1 |
| 19:50138023:A:T | rs6509441 | 19 | 50138023 | 3.30E-08 | 1 |
| 20:22245008:G:T | rs1415802 | 20 | 22245008 | 4.76E-09 | 1 |
| 20:31099311:C:T | rs159428 | 20 | 31099311 | 6.97E-09 | 1 |
| 20:47411456:C:T | rs2208590 | 20 | 47411456 | 7.81E-10 | 8 |
| 20:47723127:C:G | rs2426132 | 20 | 47723127 | 3.57E-21 | 15 |
| 21:40516070:A:G | rs2836921 | 21 | 40516070 | 4.21E-08 | 1 |
| 22:27255690:A:G | rs136554 | 22 | 27255690 | 3.53E-08 | 1 |
| 22:38138379:C:G | rs4396807 | 22 | 38138379 | 2.77E-08 | 1 |
| 22:39834102:C:G | rs73167342 | 22 | 39834102 | 2.30E-12 | 3 |
| 22:39874314:A:C | rs2899319 | 22 | 39874314 | 7.20E-11 | 4 |
| 22:40828375:A:T | rs8139974 | 22 | 40828375 | 1.52E-08 | 1 |
| 22:41461836:A:G | rs4821995 | 22 | 41461836 | 1.28E-09 | 5 |
| 22:41992169:A:G | rs62236533 | 22 | 41992169 | 5.24E-14 | 10 |
| 22:42079361:A:G | rs148537066 | 22 | 42079361 | 7.25E-09 | 2 |
| 22:42370991:C:T | rs5751191 | 22 | 42370991 | 8.93E-15 | 7 |
| 22:42717590:A:G | rs2413685 | 22 | 42717590 | 2.92E-08 | 2 |
