# Supplementary material for: Causal relationship and shared genetic pathways between diabetic kidney disease and cognitive impairment: a Mendelian randomization study
Source: Ren Fail. 2025 Jul 1;47(1):2525471. doi: 10.1080/0886022X.2025.2525471 (PMC12217110; doi:10.1080/0886022X.2025.2525471)
Supplement: Supplementary Table 2.docx [file IRNF_A_2525471_SM3598.docx]

**Supplementary Table 2. Details of the confounders in the analysis**

| **Confounder** | **ID** | **Sample size** |
| --- | --- | --- |
| Alzheimer's disease | finn-b-G6 ALZHEIMER | 218,792 |
| Heart failure | ebi-a-GCST009541 | 977,323 |
| Hypertension | ukb-b-12493 | 463,010 |
| Anxiety | ukb-b-18336 | 460,702 |
| Depression | ukb-a-81 | 337,159 |
| Education | ieu-a-755 | 106,736 |
| Smoking | ieu-b-24 | 341,427 |
| Alcohol consumption | ukb-a-25 | 336,965 |
| Income | ukb-b-7408 | 397,751 |
