# Supplementary material for: Causal relationship and shared genetic pathways between diabetic kidney disease and cognitive impairment: a Mendelian randomization study
Source: Ren Fail. 2025 Jul 1;47(1):2525471. doi: 10.1080/0886022X.2025.2525471 (PMC12217110; doi:10.1080/0886022X.2025.2525471)
Supplement: Supplementary Table 5.docx [file IRNF_A_2525471_SM3596.docx]

**Supplementary Table 5. Heterogeneity test results for Mendelian randomization analyses**

| **exposure** | **outcome** | **method** | **egger intercept** | **SE** | **pvalue** |
| --- | --- | --- | --- | --- | --- |
| DM Nephropathy | Cognition | MR-Egger | -0.010 | 0.003 | 0.096 |
|  |  | MR MRPRESSO | NA | NA | 0.147 |
| DM Nephropathy exmore |  | MR-Egger | -0.004 | 0.003 | 0.155 |
|  |  | MR MRPRESSO | NA | NA | 0.316 |
| DM1REN |  | MR-Egger | -0.064 | 0.044 | 0.218 |
|  |  | MR MRPRESSO | NA | NA | 0.956 |
| DM2REN |  | MR-Egger | 0.007 | 0.009 | 0.409 |
|  |  | MR MRPRESSO | NA | NA | 0.589 |
