# Supplementary material for: Causal relationship and shared genetic pathways between diabetic kidney disease and cognitive impairment: a Mendelian randomization study
Source: Ren Fail. 2025 Jul 1;47(1):2525471. doi: 10.1080/0886022X.2025.2525471 (PMC12217110; doi:10.1080/0886022X.2025.2525471)
Supplement: Supplementary Table 6.docx [file IRNF_A_2525471_SM3595.docx]

**Supplementary Table 6. Steiger directionality test results for diabetic kidney disease and cognitive impairment causal inference**

| **SNP** | **exposure** | **outcome** | **rsq.exposure** | **rsq.outcome** | **steiger_dir** | **steiger_pval** |
| --- | --- | --- | --- | --- | --- | --- |
| rs1055348 | DM_Nephropathy_exmore | Cognition | 9.13E-05 | 3.61E-07 | TRUE | 9.31E-07 |
| rs1055890 | DM_Nephropathy_exmore | Cognition | 1.49E-04 | 1.48E-06 | TRUE | 1.62E-09 |
| rs115368838 | DM_Nephropathy_exmore | Cognition | 7.96E-05 | 1.36E-06 | TRUE | 2.16E-05 |
| rs115641163 | DM_Nephropathy_exmore | Cognition | 1.91E-04 | 2.80E-06 | TRUE | 2.73E-11 |
| rs118143045 | DM_Nephropathy_exmore | Cognition | 1.71E-04 | 2.24E-06 | TRUE | 2.34E-10 |
| rs12055445 | DM_Nephropathy_exmore | Cognition | 6.44E-04 | 5.33E-06 | TRUE | 1.23E-36 |
| rs1265097 | DM_Nephropathy_exmore | Cognition | 9.57E-05 | 1.49E-07 | TRUE | 2.64E-07 |
| rs144092339 | DM_Nephropathy_exmore | Cognition | 1.75E-04 | 1.50E-07 | TRUE | 1.87E-12 |
| rs144580787 | DM_Nephropathy_exmore | Cognition | 9.18E-05 | 2.88E-07 | TRUE | 7.25E-07 |
| rs151102334 | DM_Nephropathy_exmore | Cognition | 9.60E-05 | 1.07E-08 | TRUE | 1.10E-07 |
| rs17201144 | DM_Nephropathy_exmore | Cognition | 7.75E-05 | 5.21E-07 | TRUE | 9.63E-06 |
| rs17220087 | DM_Nephropathy_exmore | Cognition | 1.06E-04 | 3.46E-09 | TRUE | 1.93E-08 |
| rs1894406 | DM_Nephropathy_exmore | Cognition | 6.42E-04 | 2.26E-06 | TRUE | 5.36E-39 |
| rs2071463 | DM_Nephropathy_exmore | Cognition | 1.06E-04 | 1.21E-08 | TRUE | 2.50E-08 |
| rs2071472 | DM_Nephropathy_exmore | Cognition | 3.85E-04 | 2.48E-06 | TRUE | 4.84E-23 |
| rs2157077 | DM_Nephropathy_exmore | Cognition | 6.22E-04 | 2.13E-06 | TRUE | 6.72E-38 |
| rs2252937 | DM_Nephropathy_exmore | Cognition | 1.95E-04 | 9.89E-08 | TRUE | 7.50E-14 |
| rs2257126 | DM_Nephropathy_exmore | Cognition | 1.51E-04 | 1.30E-05 | TRUE | 1.90E-06 |
| rs2284191 | DM_Nephropathy_exmore | Cognition | 9.03E-05 | 5.59E-06 | TRUE | 9.16E-05 |
| rs241447 | DM_Nephropathy_exmore | Cognition | 4.27E-04 | 2.49E-06 | TRUE | 1.46E-25 |
| rs2523613 | DM_Nephropathy_exmore | Cognition | 7.34E-05 | 6.01E-08 | TRUE | 5.14E-06 |
| rs2523614 | DM_Nephropathy_exmore | Cognition | 1.20E-04 | 1.72E-06 | TRUE | 1.30E-07 |
| rs28361065 | DM_Nephropathy_exmore | Cognition | 7.57E-05 | 7.86E-07 | TRUE | 1.87E-05 |
| rs28383322 | DM_Nephropathy_exmore | Cognition | 8.91E-05 | 3.67E-07 | TRUE | 1.31E-06 |
| rs28533694 | DM_Nephropathy_exmore | Cognition | 3.45E-04 | 9.67E-08 | TRUE | 1.53E-23 |
| rs28688825 | DM_Nephropathy_exmore | Cognition | 8.41E-05 | 1.88E-11 | TRUE | 5.13E-07 |
| rs403414 | DM_Nephropathy_exmore | Cognition | 1.16E-04 | 5.54E-06 | TRUE | 4.17E-06 |
| rs41266701 | DM_Nephropathy_exmore | Cognition | 1.75E-04 | 5.05E-06 | TRUE | 1.87E-09 |
| rs4148872 | DM_Nephropathy_exmore | Cognition | 1.16E-04 | 8.69E-08 | TRUE | 1.00E-08 |
| rs4380799 | DM_Nephropathy_exmore | Cognition | 1.38E-04 | 3.82E-06 | TRUE | 8.02E-08 |
| rs4713555 | DM_Nephropathy_exmore | Cognition | 1.31E-04 | 1.53E-06 | TRUE | 2.32E-08 |
| rs4947324 | DM_Nephropathy_exmore | Cognition | 1.40E-04 | 1.67E-06 | TRUE | 7.32E-09 |
| rs57493997 | DM_Nephropathy_exmore | Cognition | 8.50E-05 | 6.10E-07 | TRUE | 3.82E-06 |
| rs74618856 | DM_Nephropathy_exmore | Cognition | 2.10E-04 | 1.94E-05 | TRUE | 3.47E-08 |
| rs7762120 | DM_Nephropathy_exmore | Cognition | 9.81E-05 | 3.73E-10 | TRUE | 6.17E-08 |
| rs9266276 | DM_Nephropathy_exmore | Cognition | 2.45E-04 | 1.27E-07 | TRUE | 5.52E-17 |
| rs9380326 | DM_Nephropathy_exmore | Cognition | 1.32E-04 | 1.59E-09 | TRUE | 3.49E-10 |
| rs9405002 | DM_Nephropathy_exmore | Cognition | 2.32E-04 | 2.53E-07 | TRUE | 7.03E-16 |
| rs9469079 | DM_Nephropathy_exmore | Cognition | 1.22E-04 | 1.54E-09 | TRUE | 1.68E-09 |
| rs115641163 | DM_Nephropathy | Cognition | 1.32E-04 | 2.80E-06 | TRUE | 7.16E-08 |
| rs115892333 | DM_Nephropathy | Cognition | 7.80E-05 | 2.00E-07 | TRUE | 4.36E-06 |
| rs116015488 | DM_Nephropathy | Cognition | 1.17E-04 | 2.75E-06 | TRUE | 5.45E-07 |
| rs11753831 | DM_Nephropathy | Cognition | 8.11E-05 | 5.89E-06 | TRUE | 3.13E-04 |
| rs12055445 | DM_Nephropathy | Cognition | 3.12E-04 | 5.33E-06 | TRUE | 3.93E-17 |
| rs1894406 | DM_Nephropathy | Cognition | 3.28E-04 | 2.26E-06 | TRUE | 9.74E-20 |
| rs2071470 | DM_Nephropathy | Cognition | 1.75E-04 | 2.28E-06 | TRUE | 1.43E-10 |
| rs209473 | DM_Nephropathy | Cognition | 7.29E-05 | 6.54E-06 | TRUE | 1.05E-03 |
| rs210130 | DM_Nephropathy | Cognition | 7.28E-05 | 4.16E-06 | TRUE | 3.75E-04 |
| rs2157077 | DM_Nephropathy | Cognition | 2.90E-04 | 2.13E-06 | TRUE | 1.47E-17 |
| rs241442 | DM_Nephropathy | Cognition | 2.14E-04 | 2.41E-06 | TRUE | 7.84E-13 |
| rs2523615 | DM_Nephropathy | Cognition | 9.74E-05 | 2.00E-06 | TRUE | 3.64E-06 |
| rs28383322 | DM_Nephropathy | Cognition | 7.28E-05 | 3.67E-07 | TRUE | 1.40E-05 |
| rs2855441 | DM_Nephropathy | Cognition | 9.61E-05 | 1.02E-05 | TRUE | 2.96E-04 |
| rs2855450 | DM_Nephropathy | Cognition | 9.85E-05 | 1.23E-05 | TRUE | 4.38E-04 |
| rs3819714 | DM_Nephropathy | Cognition | 8.72E-05 | 1.15E-06 | TRUE | 5.93E-06 |
| rs403414 | DM_Nephropathy | Cognition | 7.54E-05 | 5.54E-06 | TRUE | 5.26E-04 |
| rs41266701 | DM_Nephropathy | Cognition | 1.18E-04 | 5.05E-06 | TRUE | 2.48E-06 |
| rs416622 | DM_Nephropathy | Cognition | 7.74E-05 | 4.42E-06 | TRUE | 2.46E-04 |
| rs4947324 | DM_Nephropathy | Cognition | 9.21E-05 | 1.67E-06 | TRUE | 5.35E-06 |
| rs72844760 | DM_Nephropathy | Cognition | 8.01E-05 | 2.55E-06 | TRUE | 5.62E-05 |
| rs74618856 | DM_Nephropathy | Cognition | 1.11E-04 | 1.94E-05 | TRUE | 7.53E-04 |
| rs13195402 | DM1REN | Cognition | 1.04E-04 | 6.29E-05 | TRUE | 1.48E-02 |
| rs13217620 | DM1REN | Cognition | 8.93E-05 | 6.91E-05 | TRUE | 3.47E-02 |
| rs71557378 | DM1REN | Cognition | 8.95E-05 | 6.78E-05 | TRUE | 5.02E-01 |
| rs72847313 | DM1REN | Cognition | 9.40E-05 | 6.51E-05 | TRUE | 3.73E-01 |
| rs7749305 | DM1REN | Cognition | 1.13E-04 | 5.73E-05 | TRUE | 4.56E-03 |
| rs7752448 | DM1REN | Cognition | 9.42E-05 | 6.86E-05 | TRUE | 1.67E-03 |
| rs10804330 | DM2REN | Cognition | 7.97E-05 | 5.95E-07 | TRUE | 7.95E-06 |
| rs11196175 | DM2REN | Cognition | 9.03E-05 | 4.57E-06 | TRUE | 5.50E-05 |
| rs11244 | DM2REN | Cognition | 1.29E-04 | 3.68E-06 | TRUE | 2.43E-07 |
| rs12055445 | DM2REN | Cognition | 1.14E-04 | 5.33E-06 | TRUE | 4.49E-06 |
| rs1800683 | DM2REN | Cognition | 7.69E-05 | 1.40E-05 | TRUE | 5.91E-03 |
| rs1894406 | DM2REN | Cognition | 1.03E-04 | 2.26E-06 | TRUE | 2.17E-06 |
| rs2157077 | DM2REN | Cognition | 9.41E-05 | 2.13E-06 | TRUE | 6.39E-06 |
| rs2239701 | DM2REN | Cognition | 8.29E-05 | 2.43E-07 | TRUE | 2.40E-06 |
| rs2256965 | DM2REN | Cognition | 8.58E-05 | 6.04E-08 | TRUE | 7.87E-07 |
| rs241447 | DM2REN | Cognition | 7.50E-05 | 2.49E-06 | TRUE | 1.05E-04 |
| rs2713557 | DM2REN | Cognition | 7.98E-05 | 5.49E-07 | TRUE | 7.24E-06 |
| rs9270505 | DM2REN | Cognition | 7.43E-05 | 8.25E-07 | TRUE | 2.40E-05 |
| rs9276807 | DM2REN | Cognition | 9.58E-05 | 3.82E-06 | TRUE | 1.77E-05 |
