# Supplementary material for: Causal relationship and shared genetic pathways between diabetic kidney disease and cognitive impairment: a Mendelian randomization study
Source: Ren Fail. 2025 Jul 1;47(1):2525471. doi: 10.1080/0886022X.2025.2525471 (PMC12217110; doi:10.1080/0886022X.2025.2525471)
Supplement: Supplementary Table 4.docx [file IRNF_A_2525471_SM3594.docx]

**Supplementary Table 4. Mendelian randomization analysis assessing causal associations between diabetic kidney disease and cognitive function**

| **exposure** | **outcome** | **method** | **nsnp** | **b** | **se** | **pval** | **lo_ci** | **up_ci** | **or** | **or_lci95** | **or_uci95** |
| --- | --- | --- | --- | --- | --- | --- | --- | --- | --- | --- | --- |
| DM_Nephropathy | Cognition | MR Egger | 22 | 0.103 | 0.072 | 0.236 | -0.038 | 0.244 | 1.11 | 0.96 | 1.28 |
| DM_Nephropathy | Cognition | Weighted median | 22 | -0.153 | 0.028 | ＜0.001 | -0.207 | -0.099 | 0.86 | 0.81 | 0.91 |
| DM_Nephropathy | Cognition | Inverse variance weighted | 22 | -0.202 | 0.023 | ＜0.001 | -0.246 | -0.157 | 0.82 | 0.78 | 0.85 |
| DM_Nephropathy | Cognition | Simple mode | 22 | -0.150 | 0.046 | 0.010 | -0.239 | -0.060 | 0.86 | 0.79 | 0.94 |
| DM_Nephropathy | Cognition | Weighted mode | 22 | -0.135 | 0.033 | 0.002 | -0.199 | -0.070 | 0.87 | 0.82 | 0.93 |
| DM_Nephropathy_exmore | Cognition | MR Egger | 39 | -0.021 | 0.062 | 0.774 | -0.142 | 0.100 | 0.98 | 0.87 | 1.11 |
| DM_Nephropathy_exmore | Cognition | Weighted median | 39 | -0.101 | 0.021 | ＜0.001 | -0.143 | -0.059 | 0.90 | 0.87 | 0.94 |
| DM_Nephropathy_exmore | Cognition | Inverse variance weighted | 39 | -0.122 | 0.016 | ＜0.001 | -0.153 | -0.092 | 0.88 | 0.86 | 0.91 |
| DM_Nephropathy_exmore | Cognition | Simple mode | 39 | -0.083 | 0.034 | 0.046 | -0.150 | -0.015 | 0.92 | 0.86 | 0.98 |
| DM_Nephropathy_exmore | Cognition | Weighted mode | 39 | -0.104 | 0.026 | 0.002 | -0.156 | -0.052 | 0.90 | 0.86 | 0.95 |
| DM1REN | Cognition | MR Egger | 6 | 0.416 | 0.596 | 0.582 | -0.753 | 1.585 | 1.52 | 0.47 | 4.88 |
| DM1REN | Cognition | Weighted median | 6 | -0.606 | 0.050 | ＜0.001 | -0.704 | -0.509 | 0.55 | 0.49 | 0.60 |
| DM1REN | Cognition | Inverse variance weighted | 6 | -0.600 | 0.025 | ＜0.001 | -0.648 | -0.551 | 0.55 | 0.52 | 0.58 |
| DM1REN | Cognition | Simple mode | 6 | -0.629 | 0.070 | ＜0.001 | -0.766 | -0.493 | 0.53 | 0.46 | 0.61 |
| DM1REN | Cognition | Weighted mode | 6 | -0.625 | 0.069 | ＜0.001 | -0.760 | -0.491 | 0.54 | 0.47 | 0.61 |
| DM2REN | Cognition | MR Egger | 13 | -0.483 | 0.291 | 0.182 | -1.053 | 0.086 | 0.62 | 0.35 | 1.09 |
| DM2REN | Cognition | Weighted median | 13 | -0.186 | 0.039 | ＜0.001 | -0.263 | -0.110 | 0.83 | 0.77 | 0.90 |
| DM2REN | Cognition | Inverse variance weighted | 13 | -0.193 | 0.029 | ＜0.001 | -0.250 | -0.137 | 0.82 | 0.78 | 0.87 |
| DM2REN | Cognition | Simple mode | 13 | -0.217 | 0.059 | 0.008 | -0.333 | -0.101 | 0.80 | 0.72 | 0.90 |
| DM2REN | Cognition | Weighted mode | 13 | -0.219 | 0.061 | 0.009 | -0.337 | -0.100 | 0.80 | 0.71 | 0.90 |
