# Supplementary material for: Causal relationship and shared genetic pathways between diabetic kidney disease and cognitive impairment: a Mendelian randomization study
Source: Ren Fail. 2025 Jul 1;47(1):2525471. doi: 10.1080/0886022X.2025.2525471 (PMC12217110; doi:10.1080/0886022X.2025.2525471)
Supplement: Supplementary Table 3.docx [file IRNF_A_2525471_SM3593.docx]

**Supplementary Table 3. Significant S-LDSC annotation categories enriched in cognition-related GWAS (P < 0.05, FDR-corrected)**

| **Category** | **Prop. SNPs** | **Prop. h2** | **Prop. h2 std error** | **Enrichment** | **Enrichment std error** | **Enrichment p** |
| --- | --- | --- | --- | --- | --- | --- |
| Coding UCSCL2 0 | 0.01 | 0.10 | 0.01 | 6.90 | 0.91 | 5.85E-10 |
| Coding UCSC.flanking.500L2 0 | 0.05 | 0.00 | 0.02 | 0.03 | 0.39 | 1.38E-02 |
| Conserved LindbladTohL2 0 | 0.02 | 0.20 | 0.03 | 8.13 | 1.19 | 5.80E-09 |
| FetalDHS TrynkaL2 0 | 0.08 | 0.20 | 0.04 | 2.43 | 0.53 | 7.79E-03 |
| H3K27ac HniszL2 0 | 0.39 | 0.46 | 0.02 | 1.17 | 0.05 | 1.04E-03 |
| H3K27ac PGC2L2 0 | 0.27 | 0.34 | 0.03 | 1.28 | 0.11 | 1.68E-02 |
| H3K4me1 peaks TrynkaL2 0 | 0.17 | 0.32 | 0.05 | 1.92 | 0.29 | 1.56E-03 |
| H3K4me1 TrynkaL2 0 | 0.42 | 0.56 | 0.04 | 1.32 | 0.10 | 2.09E-03 |
| H3K4me3 TrynkaL2 0 | 0.13 | 0.23 | 0.03 | 1.73 | 0.24 | 2.57E-03 |
| H3K9ac peaks TrynkaL2 0 | 0.04 | 0.11 | 0.03 | 3.00 | 0.72 | 6.39E-03 |
| H3K9ac TrynkaL2 0 | 0.13 | 0.23 | 0.03 | 1.82 | 0.22 | 3.34E-04 |
| Intron UCSCL2 0 | 0.39 | 0.43 | 0.02 | 1.10 | 0.04 | 1.79E-02 |
| Intron UCSC.flanking.500L2 0 | 0.01 | 0.07 | 0.01 | 7.33 | 1.42 | 1.61E-05 |
| Promoter UCSCL2 0 | 0.05 | 0.10 | 0.02 | 2.20 | 0.39 | 2.24E-03 |
| SuperEnhancer HniszL2 0 | 0.17 | 0.21 | 0.01 | 1.26 | 0.07 | 2.03E-04 |
| Transcr HoffmanL2 0 | 0.35 | 0.46 | 0.04 | 1.33 | 0.12 | 7.22E-03 |
| Transcr Hoffman.flanking.500L2 0 | 0.42 | 0.27 | 0.05 | 0.65 | 0.12 | 5.02E-03 |
| TSS HoffmanL2 0 | 0.02 | 0.05 | 0.02 | 2.88 | 0.92 | 4.43E-02 |
| UTR 3 UCSCL2 0 | 0.01 | 0.04 | 0.01 | 4.00 | 0.96 | 1.96E-03 |
| UTR 5 UCSCL2 0 | 0.01 | 0.02 | 0.01 | 4.22 | 1.42 | 2.45E-02 |
| WeakEnhancer HoffmanL2 0 | 0.02 | 0.06 | 0.02 | 3.09 | 0.91 | 2.24E-02 |
| GERP.NSL2 0 | 1.75 | 3.45 | 0.12 | 1.98 | 0.07 | 5.01E-34 |
| GERP.RSsup4L2 0 | 0.01 | 0.10 | 0.02 | 11.99 | 2.73 | 7.17E-05 |
| MAFbin1L2 0 | 0.10 | 0.07 | 0.01 | 0.70 | 0.09 | 1.37E-03 |
| MAFbin2L2 0 | 0.10 | 0.03 | 0.01 | 0.34 | 0.10 | 3.27E-11 |
| MAFbin3L2 0 | 0.10 | 0.07 | 0.01 | 0.72 | 0.12 | 1.64E-02 |
| MAFbin4L2 0 | 0.10 | 0.06 | 0.01 | 0.64 | 0.11 | 1.14E-03 |
| MAFbin8L2 0 | 0.10 | 0.14 | 0.02 | 1.35 | 0.17 | 4.02E-02 |
| MAFbin9L2 0 | 0.10 | 0.15 | 0.02 | 1.45 | 0.16 | 5.63E-03 |
| MAFbin10L2 0 | 0.10 | 0.16 | 0.02 | 1.64 | 0.17 | 1.74E-04 |
| MAF Adj Predicted Allele AgeL2 0 | 0.00 | -0.35 | 0.05 | -103799.76 | 16041.70 | 4.81E-09 |
| MAF Adj LLD AFRL2 0 | 0.00 | -0.31 | 0.03 | -109.42 | 11.70 | 4.00E-15 |
| Recomb Rate 10kbL2 0 | 1.55 | 1.31 | 0.09 | 0.84 | 0.06 | 4.18E-03 |
| Nucleotide Diversity 10kbL2 0 | 4.61 | 3.54 | 0.09 | 0.77 | 0.02 | 2.37E-27 |
| Backgrd Selection StatL2 0 | 0.18 | 0.22 | 0.01 | 1.26 | 0.03 | 2.76E-15 |
| CpG Content 50kbL2 0 | 0.01 | 0.01 | 0.00 | 1.09 | 0.01 | 6.92E-10 |
| MAF Adj ASMCL2 0 | 0.00 | -0.52 | 0.04 | -1.83E+15 | 1.50E+14 | 1.94E-25 |
| GTEx eQTL MaxCPPL2 0 | 0.01 | 0.04 | 0.01 | 4.12 | 0.80 | 1.16E-04 |
| synonymousL2 0 | 0.00 | 0.03 | 0.01 | 10.50 | 3.24 | 3.46E-03 |
| non synonymousL2 0 | 0.00 | 0.02 | 0.01 | 6.98 | 2.59 | 2.36E-02 |
| Conserved Vertebrate phastCons46wayL2 0 | 0.03 | 0.20 | 0.03 | 6.70 | 0.93 | 3.79E-09 |
| Conserved Mammal phastCons46wayL2 0 | 0.02 | 0.19 | 0.03 | 9.09 | 1.32 | 2.92E-09 |
| Conserved Primate phastCons46wayL2 0 | 0.02 | 0.25 | 0.03 | 12.75 | 1.68 | 1.04E-11 |
| Conserved Primate phastCons46way.flanking.500L2 0 | 0.16 | 0.26 | 0.04 | 1.68 | 0.25 | 7.52E-03 |
| BivFlnkL2 0 | 0.01 | 0.04 | 0.01 | 3.24 | 1.03 | 3.12E-02 |
| Human Promoter VillarL2 0 | 0.02 | 0.05 | 0.01 | 2.98 | 0.70 | 5.46E-03 |
| Ancient Sequence Age Human PromoterL2 0 | 0.00 | 0.03 | 0.01 | 7.33 | 2.56 | 1.49E-02 |
| Ancient Sequence Age Human Promoter.flanking.500L2 0 | 0.01 | 0.03 | 0.01 | 5.94 | 2.24 | 2.76E-02 |
| Human Enhancer Villar Species Enhancer CountL2 0 | 0.07 | 0.13 | 0.03 | 1.89 | 0.45 | 4.75E-02 |
| Human Promoter Villar ExACL2 0 | 0.00 | 0.02 | 0.01 | 6.88 | 2.17 | 7.12E-03 |
| H3K4me3 BasenjiL2 0 | 0.01 | 0.01 | 0.00 | 1.36 | 0.18 | 4.08E-02 |
| MPCL2 0 | 0.00 | 0.02 | 0.00 | 43.35 | 10.10 | 5.17E-05 |
| CADDL2 0 | 0.00 | 0.07 | 0.01 | 18.50 | 3.60 | 2.16E-06 |
| ReMML2 0 | 0.00 | 0.05 | 0.01 | 14.34 | 3.65 | 3.19E-04 |
| PrimateAI boosted.perc999L2 0 | 0.00 | 0.02 | 0.01 | 28.62 | 8.94 | 2.20E-03 |
| EigenALL boostedL2 0 | 0.04 | 0.29 | 0.04 | 6.59 | 0.95 | 7.46E-09 |
| ReMM boostedL2 0 | 0.01 | 0.13 | 0.02 | 16.65 | 2.71 | 2.69E-08 |
| ncER boostedL2 0 | 0.05 | 0.31 | 0.04 | 6.80 | 0.91 | 5.11E-10 |
| H3K9ac Trynka boostedL2 0 | 0.05 | 0.15 | 0.02 | 3.12 | 0.49 | 1.91E-05 |
| H3K9ac peaks Trynka boostedL2 0 | 0.10 | 0.23 | 0.04 | 2.36 | 0.38 | 4.43E-04 |
| zoonomia mammalsL2 0 | 0.02 | 0.11 | 0.02 | 7.52 | 1.59 | 5.57E-05 |
| zoonomia primatesL2 0 | 0.02 | 0.22 | 0.02 | 13.86 | 1.59 | 5.50E-14 |
